# *Bacillus anthracis* Peptidoglycan Integrity Is Disrupted by the Chemokine CXCL10 through the FtsE/X Complex

**DOI:** 10.3389/fmicb.2017.00740

**Published:** 2017-04-27

**Authors:** Katie R. Margulieux, Benjamin K. Liebov, Venkata S. K. K. S. Tirumala, Arpita Singh, John H. Bushweller, Robert K. Nakamoto, Molly A. Hughes

**Affiliations:** ^1^Division of Infectious Diseases and International Health, Department of Medicine, School of Medicine, University of Virginia, CharlottesvilleVA, USA; ^2^Department of Chemistry, University of Virginia, CharlottesvilleVA, USA; ^3^Department of Molecular Physiology and Biological Physics, University of Virginia, CharlottesvilleVA, USA

**Keywords:** *Bacillus anthracis*, chemokine, CXCL10, antimicrobial, FtsE/X, peptidoglycan

## Abstract

The antimicrobial activity of the chemokine CXCL10 against vegetative cells of *Bacillus anthracis* occurs via both bacterial FtsE/X-dependent and-independent pathways. Previous studies established that the FtsE/X-dependent pathway was mediated through interaction of the N-terminal region(s) of CXCL10 with a functional FtsE/X complex, while the FtsE/X-independent pathway was mediated through the C-terminal α-helix of CXCL10. Both pathways result in cell lysis and death of *B. anthracis*. In other bacterial species, it has been shown that FtsE/X is involved in cellular elongation though activation of complex-associated peptidoglycan hydrolases. Thus, we hypothesized that the CXCL10-mediated killing of vegetative cells of *B. anthracis* through the FtsE/X-dependent pathway resulted from the disruption of peptidoglycan processing. Immunofluorescence microscopy studies using fluorescent peptidoglycan probes revealed that incubation of *B. anthracis* Sterne (parent) strain with CXCL10 or a C-terminal truncated CXCL10 (CTTC) affected peptidoglycan processing and/or incorporation of precursors into the cell wall. *B. anthracis* Δ*ftsX* or *ftsE(K123A/D481N)* mutant strains, which lacked a functional FtsE/X complex, exhibited little to no evidence of disruption in peptidoglycan processing by either CXCL10 or CTTC. Additional studies demonstrated that the *B. anthracis* parent strain exhibited a statistically significant increase in peptidoglycan release in the presence of either CXCL10 or CTTC. While *B. anthracis* Δ*ftsX* strain showed increased peptidoglycan release in the presence of CXCL10, no increase was observed with CTTC, suggesting that the FtsE/X-independent pathway was responsible for the activity observed with CXCL10. These results indicate that FtsE/X-dependent killing of vegetative cells of *B. anthracis* results from a loss of cell wall integrity due to disruption of peptidoglycan processing and suggest that FtsE/X may be an important antimicrobial target to study in the search for alternative microbial therapeutics.

## Introduction

Chemokines are a large family of proteins that mediate the recruitment of leukocytes during the innate and adaptive immune response (Rossi and Zlotnik, 2000; Zlotnik and Yoshie, 2000, 2012). Interestingly, a number of chemokines have also been shown to have a direct antimicrobial effect against a wide variety of pathogens (Cole et al., 2001; Durr and Peschel, 2002; Yang et al., 2003; Crawford et al., 2009; Nguyen and Vogel, 2012; Yung and Murphy, 2012; Schutte et al., 2016). Among these chemokines, the human interferon-γ-inducible, Glu-Leu-Arg negative [ELR(-)] CXC chemokines, CXCL9, CXCL10, and CXCL11 have direct antimicrobial activity against vegetative cells of *Bacillus anthracis* (Crawford et al., 2009, 2010, 2011; Margulieux et al., 2016). *B. anthracis* is a Gram-positive, spore-forming bacterium that causes the disease anthrax (Mock and Fouet, 2001). Because recombinant human CXCL10 was the most potent against vegetative cells of *B. anthracis* (Crawford et al., 2009), we focused our attention on the antimicrobial effects of this chemokine. A screen of a transposon mutant library of *B. anthracis* Sterne strain for resistance to CXCL10 led to the identification of the bacterial protein FtsX as being important in mediating the bactericidal effect of CXCL10, a finding that was verified in a *ftsX* deletion mutant, *B. anthracis* Δ*ftsX* (Crawford et al., 2011). FtsX is the membrane component of FtsE/X, a complex that has been shown in a variety of Gram-positive, Gram-negative, and acid-fast bacterial species to be involved in activating cell wall hydrolases that impact peptidoglycan remodeling during cellular elongation (Gram-positive) or division (Gram-negative) (de Leeuw et al., 1999; Bernatchez et al., 2000; Schmidt et al., 2004; Mir et al., 2006, 2015; Reddy, 2007; Garti-Levi et al., 2008; Domínguez-Cuevas et al., 2013; Meisner et al., 2013; Bartual et al., 2014; Mavrici et al., 2014). Peptidoglycan remodeling is a tightly regulated process such that a sudden disruption in the process may result in loss of cell wall integrity and bacterial cell death. A similar phenomenon has been observed with penicillin and other beta-lactam antibiotics (Kohanski et al., 2010). Thus, FtsE/X may be an important bacterial target for development of new antimicrobial strategies (Bugg et al., 2011; Typas et al., 2011).

CXCL10 is a small 10-kD protein that has an unstructured N-terminal region, three anti-parallel β-strands, and an amphipathic C-terminal α-helix (Cole et al., 2001; Frederick and Clayman, 2001; Booth et al., 2002; Swaminathan et al., 2003). Notably, the positively charged, amphipathic α-helix has been generally thought to be responsible for the observed antimicrobial activity of the molecule because of the overall charge and structural similarity to defensins or cationic antimicrobial peptides (Brogden, 2005; Yeaman and Yount, 2007; Holdren et al., 2014). Recently, we observed that a C-terminal truncated CXCL10 (designated CTTC) retained antimicrobial activity (albeit, with somewhat less potency) against *B. anthracis* Sterne strain (hereby designated parent strain), but not against *B. anthracis* Δ*ftsX* (Margulieux et al., 2016). A second *B. anthracis* mutant strain was generated to test if a functional FtsE/X complex was required for CXCL10-mediated killing of *B. anthracis* vegetative cells. FtsE is an ATP-binding protein with conserved Walker A and B sites that are required for the hydrolysis of ATP to ADP and, in the presence of FtsX, co-localizes to the cell membrane (Arends et al., 2009; Domínguez-Cuevas et al., 2013; Sham et al., 2013; Mir et al., 2015). A mutant strain [*B. anthracis ftsE(K123A/D481N)*] was constructed with point mutations within the Walker A and B sites to eliminate its ability to hydrolyze ATP (Schmidt et al., 2004; Domínguez-Cuevas et al., 2013; Meisner et al., 2013; Mir et al., 2015; Margulieux et al., 2016). *B. anthracis ftsE(K123A/D481N)* showed intermediate resistance to CXCL10 and full resistance to CTTC, similar to results with the Δ*ftsX* strain, suggesting that the FtsE/X-dependent pathway of CXCL10-mediated killing requires an active FtsE/X complex and that FtsX alone is not sufficient (Margulieux et al., 2016). Results of membrane depolarization studies showed that CXCL10 caused membrane disruption of the *B. anthracis* parent and Δ*ftsX* strains; in contrast, CTTC caused membrane depolarization only in the parent strain and had no effect on the Δ*ftsX* strain (Margulieux et al., 2016). Thus, CXCL10 acts as a bifunctional antimicrobial molecule against vegetative cells of *B. anthracis* through FtsE/X-dependent and -independent mechanisms (Margulieux et al., 2016). The FtsE/X-dependent mechanism requires the N-terminal region of CXCL10; whereas, the FtsE/X-independent pathway is mediated through the C-terminal α-helix and/or other structural regions of CXCL10.

While FtsE/X has been identified as being important for the CXCL10 killing of *B. anthracis*, the mechanism of FtsE/X-dependent killing remains unknown. Peptidoglycan processing is an essential function for bacterial growth and cell division, and a disruption in peptidoglycan processing has a detrimental effect on the cell wall, leading to cell death (Vollmer and Seligman, 2010; Bugg et al., 2011; Reith and Mayer, 2011; Typas et al., 2011; Lovering et al., 2012; Vollmer, 2012; Sobhanifar et al., 2013). FtsE/X has been shown to be involved in peptidoglycan remodeling (Garti-Levi et al., 2008; Domínguez-Cuevas et al., 2013; Meisner et al., 2013). Thus, an inhibition or disruption of the complex by CXCL10 may interfere with peptidoglycan processing, resulting in a loss of cell integrity and cell death. Recent advances in peptidoglycan imaging technology allows for a more detailed study into how peptidoglycan remodeling is affected in the presence of antimicrobial molecules (Kuru et al., 2012, 2015; Liechti et al., 2014; Boersma et al., 2015). This new technology was used to study the antimicrobial effect of CXCL10 on peptidoglycan processing in *B. anthracis* vegetative cells. To assess peptidoglycan incorporation or release, we used two separate, yet complementary methods. One method utilized fluorescence confocal microscopy to visualize the uptake and incorporation of fluorescent-labeled amino acids into the peptidoglycan. The second method utilized a mammalian NF-κβ-based reporter cell line that overexpressed NOD2 to measure the release of peptidoglycan subunits. Our findings indicate that the interaction of CXCL10 with FtsX resulted in an increase in peptidoglycan release mediated via the FtsE/X-dependent pathway, which contributes to cell lysis through the loss of structural integrity.

## Materials and Methods

### Bacterial Strains and Culture Conditions

*Bacillus anthracis* Sterne strain 7702 (pXO1^+^, pXO2^-^, American Type Culture Collection, Manassas, VA, USA) was used in these experiments and designated as parent strain. *B. anthracis* Δ*ftsX* and *B. anthracis ftsE(K123A/D481N)* were derived from the Sterne strain as previously described (Crawford et al., 2011; Margulieux et al., 2016). Vegetative cells were prepared by inoculating *B. anthracis* parent strain spore stocks or *B. anthracis* mutant strain vegetative cell frozen stocks into 10 ml of brain heart infusion (BHI) broth (Difco, Franklin Lakes, NJ, USA) and incubating overnight at 37°C with shaking (250 rpm). Mid-log phase cultures were prepared the following day by diluting the overnight culture 1:20 in fresh BHI broth followed by incubation at 37°C with shaking for ∼2 h until the culture reached an optical density at 600 nm (OD_600_) between 0.6 and 0.65, at which time the cells were used for experiments. All laboratory work involving *B. anthracis* Sterne or Sterne-derived strains was approved through the University of Virginia Institutional Biosafety Committee. Biosafety level 2 practices were used for all work involving *B. anthracis* Sterne or Sterne-derived strains.

### Single Labeling of Peptidoglycan with EDA

Recent advances in the field of peptidoglycan labeling have resulted in the development of a variety of fluorescent probes for amino acids that are incorporated into the peptide side chain of peptidoglycan during cell growth and division (Kuru et al., 2012, 2015; Popham, 2013; Liechti et al., 2014, 2016; Boersma et al., 2015). Ethynyl-D-alanine (EDA) is a D-alanine molecule with an additional alkyne group that can be “clicked” together, using the principles of “click” chemistry, with fluorophores containing a reactive azide group, through a copper-mediated reaction, resulting in a stable, coupled conjugate (Kolb et al., 2001).

EDA [(S)-2-Amino-4-pentynoic acid] (Boaopharma, Inc., Natick, MA, USA) was dissolved in H_2_O and used at a concentration of 1 mM. EDA was used in conjunction with the Click-iT^®^ Cell Reaction Buffer Kit (Invitrogen, Carlsbad, CA, USA) and ‘clickable’ Alexa Fluor 488 Azide (Invitrogen, Carlsbad, CA, USA) per manufacturer’s instructions. Overnight bacterial cultures of *B. anthracis* parent strain, Δ*ftsX* strain, or *ftsE(K123A/D481N)* strain were diluted 1:20 in fresh BHI broth followed by a 1 h incubation at 37°C with shaking at 250 rpm. After 1 h, 100 mM EDA stock solution was added to the culture to a final concentration of 1 mM, and incubation was continued until an OD_600_ of between 0.6 and 0.65 was reached. The entire culture was centrifuged for 5 min at 3600 rpm to collect a pellet of the log-phase bacteria. The supernatant was discarded, and a total volume of 5 μl of the resulting pellet was placed into 500 μl Dulbecco’s modified Eagle’s medium (DMEM) (Gibco-Invitrogen, Carlsbad, CA, USA) + 10% fetal bovine serum (FBS) + 1 mM EDA and incubated for 1 h at 37°C in 5% CO_2_. After 1 h incubation with EDA, the bacterial culture was centrifuged for 8 min at 15,500 rpm, and the supernatant was discarded. A 1 ml volume of ice-cold 70% ethanol was added to the cell pellet and incubated for 1 h on ice. The ethanol solution was removed and the bacterial cells were resuspended and incubated for 1 h at room temperature in 1 ml PBS containing 3% BSA. Cells were then incubated for 1 h at room temperature in 0.5 ml of Click-iT^®^ Cell Reaction Buffer according to the manufacturer’s directions with Alexa Fluor 488 Azide (ThermoFisher Scientific, Waltham, MA, USA) to fluorescently label the EDA incorporated into the cell wall peptidoglycan. The bacterial cells were re-suspended in PBS and placed on glass slides with a coverslip and imaged using a Zeiss LSM 880 confocal microscope (Zeiss, Oberkochem, Germany) using 488 nm (Alexa Fluor 488) wavelength filter at 63X magnification.

### Dual Labeling of Peptidoglycan with EDA and TDL

TAMRA-D-lysine (TDL) was synthesized according to the protocol described previously (Kuru et al., 2012, 2015). Briefly, 5-carboxytetramethylrhodamine succinimidyl ester (Anaspec, Fremont, CA, USA) and *N*-α-BocD-Lys-OH (Chem-Impex, Bensenville, IL, USA) were mixed to form TDL. The product was purified using reverse phase HPLC on a Waters ZQ 2000 LCMS system (Waters, Milford, MA, USA) with a gradient of acetonitrile in water with 0.1% (vol/vol) formic acid 5–95% (vol/vol) acetonitrile for 20 min, eluting at 20 ml min^-1^. The two isomers were eluted on a Waters Sunfire Prep C18 OBD column with a 5 μm particle size, 19 mm internal diameter and length of 100 mm (Waters, Milford, MA, USA, product number 186008153). The solution containing the purified product was then lyophilized overnight to form a stable TDL powder, which was stored at -20°C and protected from light.

To perform dual labeling experiments, overnight cultures were diluted 1:20 in fresh BHI broth followed by a 1 h incubation at 37°C with shaking at 250 rpm. After 1 h, 100 mM EDA stock solution was added to the culture to a final concentration of 1 mM, and incubation was continued until an OD_600_ of between 0.6 and 0.65 was reached. The entire culture was centrifuged for 5 min at 3600 rpm to collect a pellet of the log-phase bacteria. The supernatant was discarded, and a total volume of 5 μl of the resulting pellet was placed into 500 μl DMEM (Gibco-Invitrogen, Carlsbad, CA, USA) + 10% FBS + 1 mM EDA and incubated for 1 h at 37°C in 5% CO_2_. After this 1 h incubation with EDA, the bacterial culture was centrifuged for 8 min at 15,500 rpm, and the supernatant discarded. A total volume of 5 μl of the resulting EDA-labeled pellet was placed into 500 μl DMEM + 10% FBS + 0.5 mM TDL, -/+ penicillin (35 μg/ml), tetracycline (40 μg/ml), CXCL10 (1.4 μM), or CTTC (2.8 μM) and incubated 1 h at 37°C in 5% CO_2_. An aliquot of each sample was transferred to individual 1.5 ml Eppendorf tubes and centrifuged for 8 min at 15,500 rpm after which the supernatant was discarded and the samples were processed and fixed for imaging as described above.

The bacterial cells were re-suspended in PBS and placed on glass slides with a coverslip and imaged using a Zeiss LSM 880 confocal microscope (Zeiss, Oberkochem, Germany) using 488 nm (Alexa Fluor 488) and 514 nm (TDL) wavelength filters at 63X magnification. Phase contrast light microscopy images and fluorescence images were collected using settings, including brightness and contrast, which were optimized and fixed based on the corresponding untreated control group at the beginning of the acquisition session. Further analyses were completed using Image J (Schneider et al., 2012). Relative fluorescent intensity was calculated as a percent control of the untreated sample. Ten individual fields per experimental run were analyzed to determine mean fluorescence intensity +/- standard deviation, as compared to results for untreated control sample of the same strain. Control and test samples were not compared between runs to account for experimental variation in the percent control determination.

### Peptidoglycan Release Assay

Overnight cultures were diluted 1:20 in fresh BHI broth followed by a 2 h incubation at 37°C with shaking at 250 rpm. Vegetative cells were diluted to approximately 5.0 × 10^6^ CFU ml^-1^ in fresh DMEM with high glucose, no glutamine, no phenol red (Gibco-Invitrogen, Carlsbad, CA, USA) that also contained 4 mM GlutaMAX^TM^ supplement, 10% (v/v) FBS (HyClone, Logan, UT, USA), and the concentration of penicillin, CXCL10, or CTTC to be tested. Aliquots of 400 μl were placed into 48 well plates and incubated at 37°C with 5% CO_2_ for 0 or 2 h. Penicillin (0.5 μg ml^-1^) was used as a positive control. CXCL10 was tested at a concentration of 1.4 μM, and CTTC was tested at 2.8 μM. At time 0 h or after a 2 h incubation, the supernatants for each sample group were collected and transferred into 1.5 ml Eppendorf tubes and centrifuged for 5 min at 15,000 rpm. The resulting supernatant was sterilized by filtration through a 13 mm 0.22 μM filter (ThermoFisher Scientific, Waltham, MA, USA) and then heat-inactivated for 5 min at 95°C. Concurrent CFU ml^-1^ measurements were determined for each sample. The HEK-Blue^TM^ hNOD2 mammalian cells (Invivogen, San Diego, CA, USA) were grown and maintained in DMEM medium containing 10% FBS, 50 U/ml penicillin, 50 μg/ml streptomycin, 100 μg/ml Normocin, 30 μg/ml blasticidin, and 100 μg/ml Zeocin at 37°C with 5% CO_2_ for up to no more than 10 passages and used as per manufacturer’s instructions. Briefly, the cells were sub-cultured into 96-well plates at a concentration of 50,000 cells well^-1^ in 180 μl total volume and were incubated with 20 μl of the filtered supernatant in triplicate for 20 h at 37°C with 5% CO_2_. Finally, 20 μl of the resulting HEK-Blue^TM^ hNOD2 supernatant was added to 180 μl HEK-Blue^TM^ detection medium (Invivogen, San Diego, CA, USA) and incubated for 24 h at 37°C with 5% CO_2_. Muramyl dipeptide (MDP) (Invivogen, San Diego, CA, USA) at a final concentration of 15 ng/ml reconstituted as a stock solution of 0.1 mg/ml in pure H_2_O was used as a positive control. Filtered DMEM was used as a medium control and pure H_2_O was used as a second negative control. The colorimetric change of the detection medium [due to secreted embryonic alkaline phosphatase (SEAP) enzyme released upon NF-κB reporter system activation] was measured at OD_650_ using an ELx800 Absorbance Reader (BioTek, Winooski, VT, USA).

### Statistical Analyses

Statistical analyses were performed using GraphPad Prism 6.0 software. Experimental groups were analyzed using an unpaired, two-tailed Student’s *t-*test or Mann–Whitney test as noted in the text or figure legends. Significant differences were determined to have a *p*-value of ≤ 0.05.

## Results

### *B. anthracis* Δ*ftsX* and *ftsE(K123A/D481N)* Exhibited Significant Morphological Differences Compared to the Parent Strain

**FIGURE 1:**
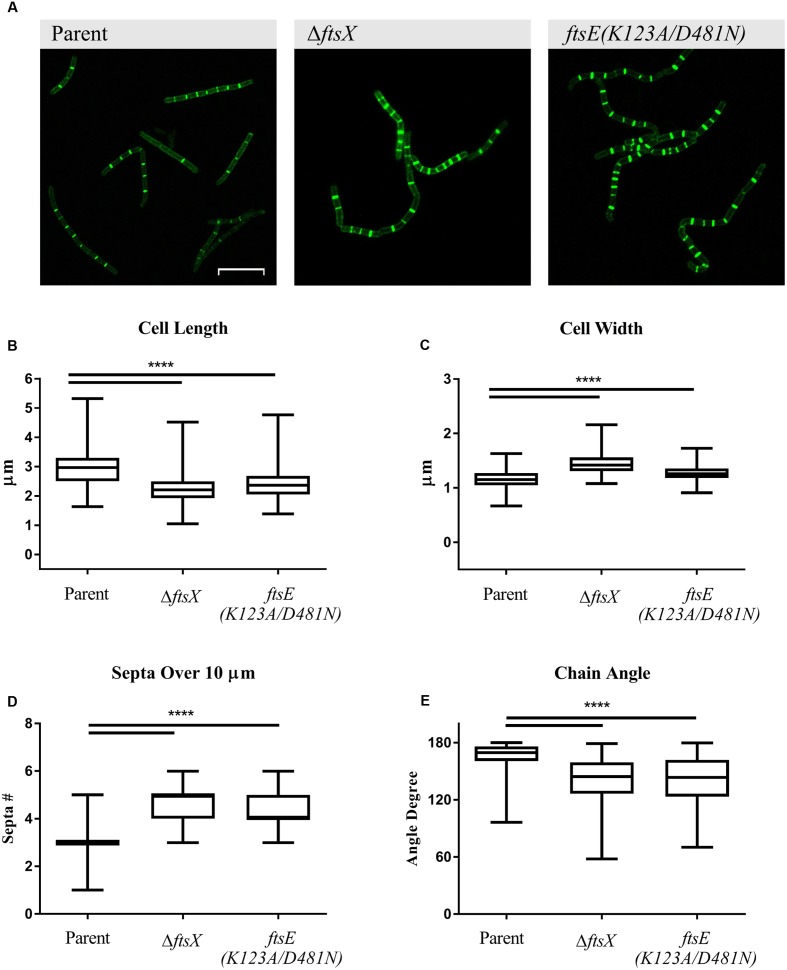
**Quantification of *Bacillus anthracis* parent strain, Δ*ftsX*, or *ftsE(K123A/D481N)* vegetative cell morphological features of cell length, width, number of septa in a given distance, and angles formed by bacterial chains.** **(A)** *B. anthracis* parent strain, *B. anthracis* Δ*ftsX* strain, and *B. anthracis ftsE(K123A/D481N)* strain visualization of incorporated EDA after “Click-iT” reaction using Alexa Fluor 488 azide. Comparison of **(B)** individual cell length, measured as the distance from a septum to an adjacent septum in a bacterial chain, **(C)** individual cell width, measured at the midpoint of the bacterial rod, **(D)** total number of septa in a bacterial chain, measured over an arbitrarily chosen distance of 10 μm, and **(E)** chain angle, as measured from end-middle-end of a chain of bacterial cells. Confocal microscopy images of untreated *B. anthracis* parent, Δ*ftsX*, and *ftsE(K123A/D481N)* strains are shown as EDA+Alexa Fluor 488 green fluorescence images at 63X magnification. Scale bar represents 10 μm. Images are representative of *n* = 3 separate experiments with 10–20 images per experiment acquired per strain. Comparisons were made between *B. anthracis* parent strain, Δ*ftsX*, and *ftsE(K123A/D481N)*. Measurements of individual vegetative cells and bacterial chains were determined using Image J (Schneider et al., 2012). A total of 100 measurements was collected each for bacterial length and width for a given strain and condition per experiment. A total of 50 measurements was collected for determining the number of septa in an arbitrarily chosen distance of 10 μm and for chain angle determination for each strain and condition per experiment. Results are represented as a box-and-whisker plot with data points represented from minimum to maximum; *n* = 3 separate experiments from which data was collected. Data were analyzed using a Mann–Whitney test, ^∗∗∗∗^*p* < 0.0001.

Previous studies with *B. anthracis* Δ*ftsX* and *ftsE(K123A/D481N)* showed that they have about a 30 min lag phase prior to initiation of log phase growth in BHI broth media compared to the parent strain (Crawford et al., 2011; Margulieux et al., 2016). However, their growth rates were similar once log phase was achieved, so all of the following experiments were conducted with mid-log phase cultures. All *B. anthracis* strains used in this study exhibited marked incorporation of EDA into the septa after 2 h of incubation, indicative of active cellular division. EDA incorporation was also noted along the cell wall of each vegetative cell, suggesting that the modified D-alanine was incorporated into the cell wall of the growing/dividing bacteria (**Figure 1A**). Notably, significant morphological differences in cell length, cell width, number of septa in a given distance, and bacterial chain angle were observed between the *B. anthracis* parent strain and the Δ*ftsX* and *ftsE(K123A/D481N)* strains (**Figures 1A–E**). In our present study, *B. anthracis* parent strain appeared as regular, even, and smooth chains of vegetative cells while the mutant strains showed a “kinked” phenotype (**Figure 1A**). Cells of *B. anthracis* Δ*ftsX* and *ftsE(K123A/D481N)* were significantly shorter in length (measured as the septum-to-septum distance of bacterial cells in a chain) and wider (measured as the width across the midpoint of the cell) compared to the parent strain (**Figures 1B,C**). These morphological differences were consistent with results reported for *Bacillus subtilis* mutants of the FtsE/X complex (Garti-Levi et al., 2008; Domínguez-Cuevas et al., 2013; Meisner et al., 2013). *B. anthracis* Δ*ftsX* and *ftsE(K123A/D481N)* also exhibited an increased number of septal division points over the arbitrarily chosen distance of 10 μm, which suggested an altered or dysregulated process of cellular division and/or elongation in these mutants (**Figures 1A,D**). The “kinked” morphology, noted above, was quantified through measurement of vegetative cell chain angles from end-middle-end of the entire chain. These measurements indicated that, compared to the *B. anthracis* parent strain, the *B. anthracis* Δ*ftsX* and *ftsE(K123A/D481N)* mutant strains exhibited a wide array of more acute angles within chains of bacteria (**Figure 1E**).

### Effects of CXCL10 and CTTC on Existing Peptidoglycan Integrity and New Incorporation of Peptidoglycan Precursors in *B. anthracis* Parent Strain

**FIGURE 2:**
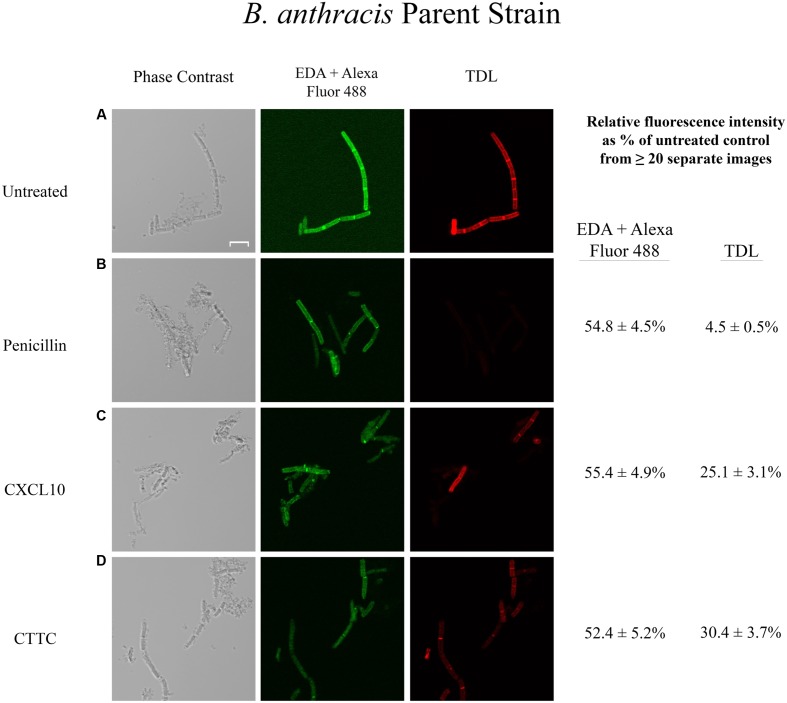
**Effects of various antimicrobial agents on the peptidoglycan of *B. anthracis* parent strain.** *B. anthracis* parent strain was treated with **(A)** buffer control, **(B)** penicillin (35 μg/ml), **(C)** CXCL10 (2.8 μM), or **(D)** CTTC (2.8 μM) to determine the effect on existing peptidoglycan integrity (EDA + Alexa Fluor 488 green fluorescence) and new subunit incorporation (TDL red fluorescence). Vegetative cells were incubated with EDA for 1 h before the addition of an antimicrobial agent (or buffer control) concurrently with TDL for 1 h, followed by a “Click-iT” reaction to bind Alexa Fluor 488 to EDA, as described in Section “Materials and Methods.” Scale bar is 5 μm. *n* = 2 separate experiments with 5–20 images being acquired for each treatment group in each experiment. Relative fluorescence intensity was determined as a percentage of the untreated control sample of the same strain.

In order to examine if the antimicrobial effect of CXCL10 and CTTC against vegetative cells of *B. anthracis* was due to an alteration of peptidoglycan integrity and/or inhibition of new synthesis, we performed dual labeling experiments using EDA and TDL as described in Section “Materials and Methods” to determine if there was a difference in peptidoglycan remodeling. Dual labeling allowed us to determine effects on existing peptidoglycan (EDA-labeled) and new peptidoglycan subunit incorporation (TDL-labeled) in the absence or presence of CXCL10, CTTC, or other antimicrobial agents. We used an untreated sample to standardize the baseline microscopy settings for the treated samples. Images of the untreated and treated sample groups for one bacterial strain were obtained in one session to ensure comparative fluorescent levels. The sequential incorporation of EDA and TDL into the septa of vegetative cells was observed with the untreated parent strain (**Figure 2A**). Penicillin was used as a positive control as it is a beta-lactam antibiotic that targets the bacterial cell wall and blocks the final cross-linking step, resulting in a loss of peptidoglycan integrity and cell lysis (**Figure 2B**) (Tipper and Strominger, 1965; Wise and Park, 1965; Novak et al., 2000). Tetracycline was used as a negative control as it is a bacteriostatic agent that inhibits protein synthesis by targeting the bacterial aminoacyl-tRNAs and 30S ribosome complex (Chopra and Roberts, 2001). In the presence of penicillin (35 μg/ml), the parent strain exhibited reduced fluorescence signal from previously incorporated EDA (54.8 + 4.5% of untreated control) and an absence (4.5 + 0.5% of untreated control) of newly incorporated TDL-labeled peptidoglycan subunits (**Figure 2B**). In contrast, incubation of *B. anthracis* parent strain with tetracycline (40 μg/ml) had a lesser impact on the incorporation of both EDA (71.0 + 3.4% of untreated control) and TDL (54.7 + 3.6% of untreated control) into the peptidoglycan (data not shown), which likely reflected that this bacteriostatic drug inhibited bacterial growth during the incubation period. In the presence of CXCL10 (1.4 μM), the *B. anthracis* parent strain exhibited decreased levels of EDA-labeled peptidoglycan (55.4 + 4.9% of untreated control) and reduced levels of TDL-labeled peptidoglycan (25.1 + 3.1% of untreated control) (**Figure 2C**). Incubation of *B. anthracis* parent strain with CTTC (2.8 μM) resulted in a similar loss of EDA and TDL fluorescence, 52.4 + 5.2% and 30.4 + 3.7% of untreated control, respectively (**Figure 2D**).

### Effects of CXCL10 and CTTC on Peptidoglycan Turnover/Synthesis of *B. anthracis* Δ*ftsX* and *ftsE(K123A/D481N)*

**FIGURE 3:**
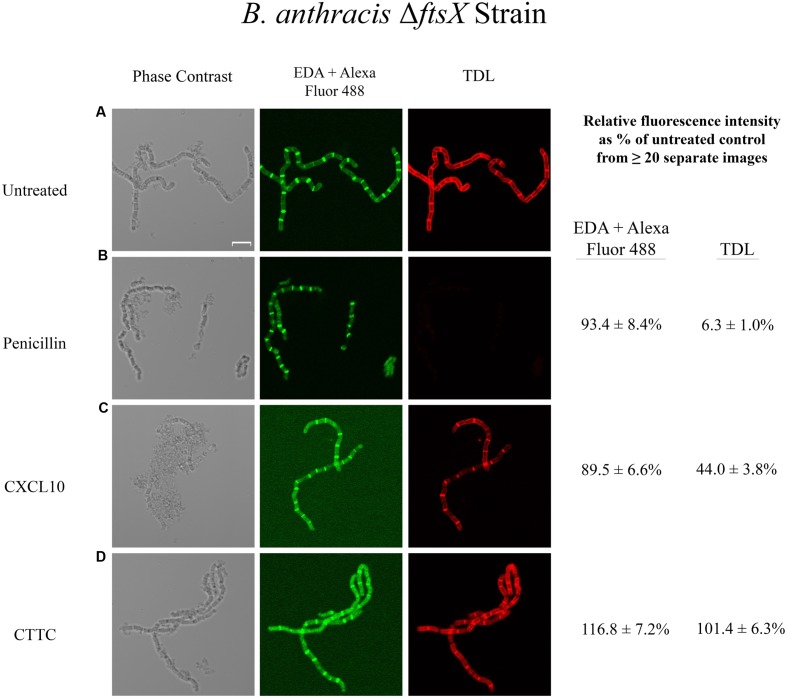
**Effects of various antimicrobial agents on the peptidoglycan of *B. anthracis* Δ*ftsX*.** *B. anthracis* Δ*ftsX* strain was treated with **(A)** buffer control, **(B)** penicillin (35 μg/ml), **(C)** CXCL10 (2.8 μM), or **(D)** CTTC (2.8 μM) to determine the effect on existing peptidoglycan integrity (EDA + Alexa Fluor 488 green fluorescence) and new subunit incorporation (TDL red fluorescence). Vegetative cells were incubated with EDA for 1 h before the addition of an antimicrobial agent (or buffer control) concurrently with TDL for 1 h, followed by a “Click-iT” reaction to bind Alexa Fluor 488 to EDA, as described in Section “Materials and Methods.” Scale bar is 5 μm. *n* = 2 separate experiments with 5–20 images being acquired for each treatment group in each experiment. Relative fluorescence intensity was determined as a percentage of the untreated control sample of the same strain.

*Bacillus anthracis* Δ*ftsX* is less susceptible to CXCL10 than the parent strain and is completely resistant to CTTC (Margulieux et al., 2016). Killing of the *B. anthracis* Δ*ftsX* strain by higher concentrations of CXCL10 appears to occur by an FtsE/X-independent pathway through general membrane depolarization and disruption via the C-terminal amphipathic α-helix of CXCL10 (Margulieux et al., 2016). The data obtained with the *B. anthracis* parent strain indicates that CXCL10 impacts peptidoglycan processing via the FtsE/X-dependent pathway; however, the *B. anthracis* Δ*ftsX* mutant lacks the FtsX target necessary for interaction with CXCL10. Thus, the only pathway by which CXCL10 can exert its effect is the previously described FtsE/X-independent mechanism that results in cellular lysis through generalized membrane depolarization. The *B. anthracis* Δ*ftsX* untreated control strain incorporated both EDA and TDL into the peptidoglycan (**Figure 3A**). Penicillin treatment resulted in an absence of TDL incorporation (6.3 + 1.0% of untreated control), i.e., newly synthesized peptidoglycan (**Figure 3B**). Notably, the EDA fluorescence (93.4 + 10.3% of untreated control) was unaffected by penicillin treatment compared to the penicillin-treated parent strain (**Figure 2B**), which suggested a possible difference between these strains to the effects of penicillin. *B. anthracis* Δ*ftsX* incubation with tetracycline gave similar results to those observed with the parent strain (data not shown). Incubation of *B. anthracis* Δ*ftsX* with CXCL10 resulted in CXCL10 having only a minor effect on the integrity of the existing (pre-treatment) peptidoglycan based on the fluorescence intensity due to EDA (89.5 + 6.6% of untreated control) and an intermediate effect on newly synthesized peptidoglycan based on reduction in TDL signal intensity (44.0 + 3.8% of untreated control) (**Figure 3C**). Under light microscopy, vegetative cells of Δ*ftsX* exhibited some cell lysis, perhaps due to membrane depolarization and cell death via the FtsX-independent pathway, which may have had some effect on fluorescence intensity compared to the untreated Δ*ftsX* control. CTTC treatment had no impact on EDA (116.8 + 7.2% of untreated control) or TDL (101.4 + 6.3% of untreated control) incorporation/uptake into the peptidoglycan of *B. anthracis* Δ*ftsX* (**Figure 3D**). These data suggested that in the absence of FtsX, the N-terminal region of CXCL10 was not able to initiate a bactericidal effect through disruption of peptidoglycan integrity or synthesis.

**FIGURE 4:**
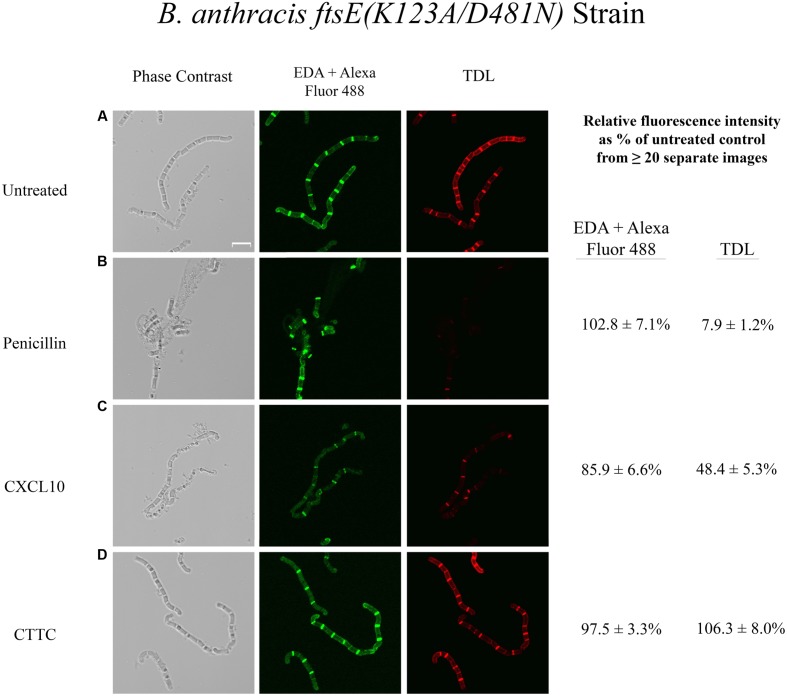
**Effects of various antimicrobial agents on the peptidoglycan of *B. anthracis ftsE(K123A/D481N)*.** *B. anthracis ftsE(K123A/D481N)* strain was treated with **(A)** buffer control, **(B)** penicillin (35 μg/ml), **(C)** CXCL10 (2.8 μM), or **(D)** CTTC (2.8 μM) to determine the effect on existing peptidoglycan integrity (EDA + Alexa Fluor 488 green fluorescence) and new subunit incorporation (TDL red fluorescence). Vegetative cells were incubated with EDA for 1 h before the addition of an antimicrobial agent (or buffer control) concurrently with TDL for 1 h, followed by a “Click-iT” reaction to bind Alexa Fluor 488 to EDA, as described in Section “Materials and Methods.” Scale bar is 5 μm. *n* = 2 separate experiments with 5–20 images being acquired for each treatment group. Relative fluorescence intensity was determined as a percentage of the untreated control sample of the same strain.

An FtsE mutant *ftsE(K123A/D481N)* was previously constructed in which select point mutations in the Walker sites eliminated ATP hydrolysis rendering the FtsE/X complex functionally inactive (Margulieux et al., 2016). Like Δ*ftsX*, the *B. anthracis ftsE(K123A/D481N)* mutant strain was also found to be less susceptible to CXCL10 and completely resistant to CTTC (Margulieux et al., 2016). Dual labeling of untreated *B. anthracis ftsE(K123A/D481N)* yielded similar results for EDA and TDL incorporation (**Figure 4A**) as observed with *B. anthracis* Δ*ftsX* (**Figure 3A**). Incubation of *ftsE(K123A/D481N)* with penicillin resulted in no loss of EDA relative fluorescence intensity (102.8 + 7.1% of untreated control), but a significant reduction in newly synthesized peptidoglycan as evidenced by reduced incorporation of TDL (7.9 + 1.2% of untreated control) (**Figure 4B**). Tetracycline treatment of the *B. anthracis ftsE(K123A/D481N)* strain gave results that were similar to those obtained with Δ*ftsX* (data not shown). CXCL10 had little effect on the incorporation of EDA in *ftsE(K123A/D481N)*, while TDL incorporation (48.4 + 5.3% of control) in this mutant strain (**Figure 4C**) was similar to the results for Δ*ftsX* (**Figure 3C**). Similar to the findings for the Δ*ftsX* strain in **Figure 3**, CTTC treatment of the *ftsE(K123A/D481N)* mutant strain had no apparent impact on existing peptidoglycan integrity as measured by EDA incorporation (97.5 + 3.9% of untreated control) or on newly synthesized peptidoglycan as measured by TDL incorporation (106.3 + 8.0% of untreated control) (**Figure 4D**).

### CXCL10 and CTTC Increased Peptidoglycan Release in *B. anthracis* Parent Strain

**FIGURE 5:**
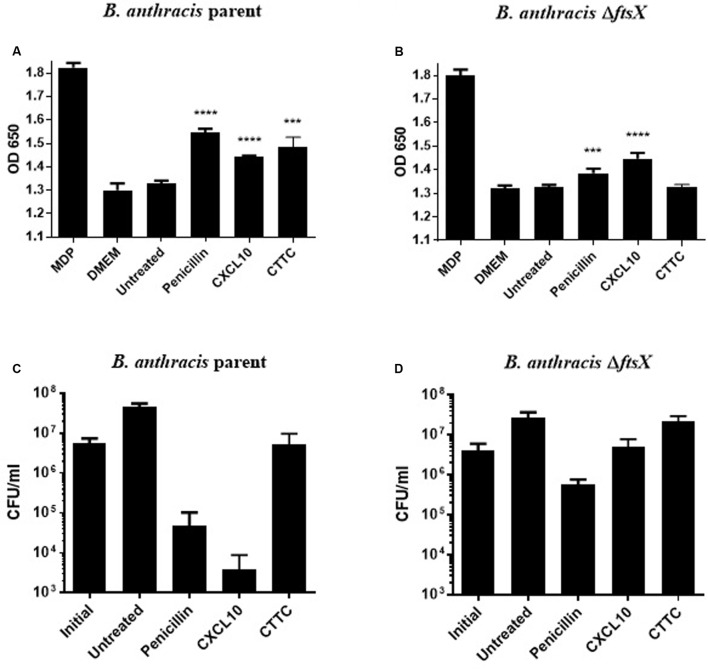
**Detection of peptidoglycan release using HEK cells that express a NOD2-NF-κB reporter system with concurrent CFU data collection.** Peptidoglycan subunit release into the supernatant of untreated or antimicrobial-treated vegetative cells was detected by an HEK-Blue^TM^ hNOD2 mammalian cell line that expresses the NOD2 receptor, which detects bacterial peptidoglycan and activates an NF-κB reporter system that produces a SEAP enzyme for a colorimetric-based quantitative readout. **(A)** Detection of *B. anthracis* parent strain peptidoglycan release in response to penicillin (0.5 μg/ml), CXCL10 (1.4 μM), or CTTC (2.8 μM), as compared to the untreated control group of the same bacterial strain. **(B)** Detection of *B. anthracis* Δ*ftsX* peptidoglycan release in response to penicillin (0.5 μg/ml), CXCL10 (1.4 μM), or CTTC (2.8 μM), as compared to the untreated control group of the same bacterial strain. CFU enumeration was performed at the 2 h time point in parallel with supernatant collection and peptidoglycan release assay. These corresponding CFU data are shown for **(C)** *B. anthracis* parent strain or **(D)** *B. anthracis* Δ*ftsX* exposed to penicillin (0.5 μg/ml), CXCL10 (1.4 μM), or CTTC (2.8 μM). Results are represented ± SEM, *n* = 3 separate experiments with triplicate wells used in each experiment. ^∗∗∗^*p* ≤ 0.0001, ^∗∗∗∗^*p* < 0.0001.

A mammalian reporter cell line that overexpressed the immune cell receptor NOD2 was used to quantitate the amount of peptidoglycan released (Packiam et al., 2015; Liechti et al., 2016). This assay provided a sensitive method for quantitating the amount of peptidoglycan released by bacteria exposed to antimicrobial agents (Girardin et al., 2003a,b; Loving et al., 2009; Philpott et al., 2014; Packiam et al., 2015). The NOD2 receptors expressed by the reporter cell line are activated by binding fragments containing N-acetylmuramic acid released from bacterial cell walls (Girardin et al., 2003a,b). The activated NOD2 receptor subsequently activates an NF-κB construct in the mammalian cell that results in the production of a SEAP reporter (Packiam et al., 2015; Liechti et al., 2016). SEAP activity causes a colorimetric change to the detection medium such that the enzymatic activity of SEAP is proportional to the amount of MDP in the original sample. An antibiotic that targets the cell wall (penicillin) was used as a positive control in this study. Tetracycline was used as a negative control. The parent strain exhibited a significant increase in peptidoglycan release, as compared to the untreated parent strain control, when exposed to penicillin (0.5 μg/ml), CXCL10 (1.4 μM), or CTTC (2.8 μM) (**Figure 5A**). In contrast, no increase in the release of peptidoglycan was observed after incubation with tetracycline (10 μg/ml) (data not shown). With the *B. anthracis* Δ*ftsX* strain, a statistically significant increase in peptidoglycan release was observed in the presence of penicillin or CXCL10 (**Figure 5B**). However, no increase in peptidoglycan release by Δ*ftsX* was observed in the presence of CTTC, suggesting that CTTC did not disrupt peptidoglycan processing. Corresponding viable bacterial cell counts (colony forming units [CFU] per ml) were also obtained for both the parent and Δ*ftsX* strains to quantitate the degree of bacterial killing under these experimental conditions (**Figures 5C,D**). Upon exposure to CXCL10, only 0.02% of the parent strain cells remained viable after 1 h while 70% of the cells in the presence of CTTC remained viable compared to the initial inoculum concentration of bacteria. Comparatively, CXCL10- or CTTC-treated samples of Δ*ftsX* were able to grow such that the CFU/ml results matched or exceeded those of the initial bacterial inoculum for the strain.

## Discussion

The FtsE/X-dependent pathway by which CXCL10 kills *B. anthracis* has been shown to be mediated through the N-terminal region(s) of CXCL10, independent of the amphipathic α-helix, but the exact mechanism of action has remained unknown. Confocal microscopy studies using fluorescence labeling of peptidoglycan amino acid components revealed that CXCL10 and CTTC caused a loss, or release, of existing peptidoglycan and decreased the incorporation of new subunits into the peptidoglycan of *B. anthracis* parent strain. The mechanism of peptidoglycan disruption by CXCL10 and CTTC appears to be distinct from that of penicillin, which was used in our studies as a cell wall active antimicrobial control. The effect of penicillin, CXCL10, and CTTC on existing peptidoglycan (EDA-labeled) was similar in all strains tested, including a similar reduction in relative fluorescence intensity from existing peptidoglycan in the *B. anthracis* parent strain that was abrogated in the Δ*ftsX* and *ftsE(K123A/D481N)* mutant strains. In contrast, penicillin blocked essentially all incorporation of newly synthesized peptidoglycan (TDL fluorescence) in each of the three strains tested, while CXCL10 and CTTC only markedly blocked TDL incorporation in the *B. anthracis* parent strain. The absence of a functional FtsE/X complex in the *B. anthracis* Δ*ftsX* and *ftsE(K123A/D481N)* mutant strains rendered CXCL10 or CTTC less effective, or completely ineffective, in inhibiting the incorporation of newly synthesized peptidoglycan.

Thus, it appears that CXCL10 and CTTC have an impact on the integrity of existing peptidoglycan and an effect on incorporation of newly synthesized peptidoglycan that is dependent on the presence of a functional FtsE/X complex. It should be noted that, in the presence of full-length CXCL10, both of the mutant strains still appeared to undergo some lysis, which was expected since CXCL10 appears to be a bifunctional antimicrobial agent that is also capable of killing *B. anthracis* through an FtsX-independent pathway (Margulieux et al., 2016). The CTTC protein, which lacks the CXCL10 amphipathic C-terminal α-helix, had no effect on peptidoglycan processing in both Δ*ftsX* and *ftsE(K123A/D481N)* mutant strains, supporting the hypothesis that a functional FtsE/X complex is required to mediate the activity of CXCL10. These observations were confirmed by measuring peptidoglycan release, which revealed that CTTC caused a release of peptidoglycan from the *B. anthracis* parent strain but was unable to cause any significant increase in release of peptidoglycan from the *B. anthracis* Δ*ftsX* mutant strain. These results support the hypothesis that FtsE/X-dependent killing by CXCL10 requires a functional FtsE/X complex and results from disruption of peptidoglycan processing.

Peptidoglycan determines cell shape and provides the structural integrity required to withstand changes in osmotic pressure (Reith and Mayer, 2011; Turner et al., 2014). Remodeling of the peptidoglycan is a vital process during all stages of the bacterial life cycle and is tightly regulated for optimal turnover and cell growth (Reith and Mayer, 2011; Lovering et al., 2012; Turner et al., 2014). The FtsE/X complex has been found to be widely conserved among Gram-positive, Gram-negative, and acid fast bacteria, and is thought to be part of a large cell wall synthesis complex that is responsible for peptidoglycan remodeling (Schmidt et al., 2004; Garti-Levi et al., 2008; Domínguez-Cuevas et al., 2013; Meisner et al., 2013; Sham et al., 2013; Bartual et al., 2014; Mavrici et al., 2014; Mir et al., 2015; Bajaj et al., 2016). Other groups have found that the FtsE/X complex in the Gram-positive bacterium *B. subtilis* is involved in cellular elongation and sporulation through the activation of peptidoglycan hydrolases that cleave old peptidoglycan bonds in order to insert new peptidoglycan subunits (Garti-Levi et al., 2008; Domínguez-Cuevas et al., 2013; Meisner et al., 2013; Mavrici et al., 2014; Mir et al., 2015). FtsE provides energy for this process through its activity as an ATP binding protein and ATP hydrolase; FtsX is a transmembrane protein that has a cell wall hydrolase-associated domain located on the external side of the cell membrane (Garti-Levi et al., 2008; Domínguez-Cuevas et al., 2013; Meisner et al., 2013; Sham et al., 2013; Mavrici et al., 2014; Mir et al., 2015). Together, FtsE and FtsX interact to form a dimerized FtsE/X complex that spans the bacterial cell membrane (Garti-Levi et al., 2008; Domínguez-Cuevas et al., 2013; Meisner et al., 2013). The hydrolysis of ATP by FtsE is thought to produce a conformational change in FtsX, which then activates and/or releases peptidoglycan hydrolases, which cleave the covalent bonds within the peptidoglycan structure (Domínguez-Cuevas et al., 2013; Meisner et al., 2013; Mavrici et al., 2014; Mir et al., 2015). This process is tightly controlled in order to maintain bacterial cell wall integrity, and any disruption, either through inhibition or overactivation of the hydrolases results in the inability to maintain the necessary internal pressure or withstand external environmental pressure (Bugg et al., 2011). CXCL10-mediated killing of *B. anthracis* via the FtsX-dependent pathway requires an active FtsE/X complex and does not simply use FtsX as a binding site for CXCL10 (Margulieux et al., 2016). FtsE/X is an intriguing antimicrobial target due to the role it has in hydrolase activation (Reith and Mayer, 2011; Vollmer, 2012; Domínguez-Cuevas et al., 2013; Meisner et al., 2013). Interestingly, in our previously published *B. anthracis* Sterne strain transposon mutant library screen, we identified two uncharacterized genes with putative lytic hydrolase domains, *BAS0651* and *lytE* (Crawford et al., 2011). The requirement for an active FtsE/X for CXCL10-mediated antimicrobial activity against *B. anthracis* and the identification of two putative hydrolases from the transposon mutant library screen raises exciting possibilities that the mechanism of action of CXCL10 and its derivatives may involve dysregulation of an FtsE/X complex-associated hydrolase (Crawford et al., 2011; Domínguez-Cuevas et al., 2013; Meisner et al., 2013; Sham et al., 2013; Bartual et al., 2014; Mavrici et al., 2014). Future studies are planned to investigate the possible role of BAS0651 and LytE, or other putative *B. anthracis* cell wall hydrolases, in the antimicrobial effect of CXCL10.

The findings presented in the current study suggest a novel mechanism for FtsE/X-dependent antimicrobial activity of CXCL10 by disrupting peptidoglycan remodeling. It appears that CXCL10 disrupts the regulation of peptidoglycan synthesis and turnover leading to an increase in peptidoglycan release and cell wall breakdown that results in cell death. FtsE/X functions in Gram-positive bacteria at the intersection of bacterial growth and homeostatic peptidoglycan remodeling, making it a promising target for new antimicrobial molecules. FtsE/X is thought to exist within a larger cell wall synthesis complex, the continued study of which will further clarify the mechanism by which CXCL10 kills vegetative cells of *B. anthracis*. These findings advance our understanding of the mechanism(s) by which CXCL10 exerts its antimicrobial effect and may ultimately facilitate development of novel antimicrobial strategies such as small molecule therapeutics derived from CXCL10 that target the FtsE/X complex.

## Author Contributions

KM, BL, VT, and AS: performed the experiments presented in this work. KM and MH: wrote the manuscript. KM, BL, VT, AS, JB, RN, and MH: designed the experiments, analyzed the resulting data, and reviewed and revised the manuscript.

## Conflict of Interest Statement

The authors declare that the research was conducted in the absence of any commercial or financial relationships that could be construed as a potential conflict of interest.
